# Cardiac Extracellular Matrix Hydrogel Enriched with Polyethylene Glycol Presents Improved Gelation Time and Increased On-Target Site Retention of Extracellular Vesicles

**DOI:** 10.3390/ijms22179226

**Published:** 2021-08-26

**Authors:** Lidia Gómez-Cid, María Luisa López-Donaire, Diego Velasco, Víctor Marín, María Isabel González, Beatriz Salinas, Lorena Cussó, Ángel García, Susana Belén Bravo, María Eugenia Fernández-Santos, Carlos Elvira, Johanna Sierra, Ester Arroba, Rafael Bañares, Lilian Grigorian-Shamagian, Francisco Fernández-Avilés

**Affiliations:** 1 Department of Cardiology, Hospital General Universitario Gregorio Marañón, 28009 Madrid, Spain; ligomezc@ing.uc3m.es (L.G.-C.); victor.magonzalez@alumnos.upm.es (V.M.); mariuge@fibhgm.org (M.E.F.-S.); francisco.fernandezaviles@salud.madrid.org (F.F.-A.); 2 Instituto de Investigación Sanitaria Gregorio Marañón, Hospital Gregorio Marañón, 28009 Madrid, Spain; divelasc@ing.uc3m.es (D.V.); migonzalez@hggm.es (M.I.G.); bsalinas@hggm.es (B.S.); lcusso@hggm.es (L.C.); johanna.sierra@iisgm.com (J.S.); ester.arroba@iisgm.com (E.A.); rbanares@ucm.es (R.B.); 3 CIBERCV, ISCIII, 28029 Madrid, Spain; 4 Departamento de Bioingeniería e Ingeniería Aeroespacial, Universidad Carlos III de Madrid, 28911 Leganés, Spain; marisalop@ictp.csic.es; 5 Unidad de Imagen Avanzada, Centro Nacional de Investigaciones Cardiovasculares (CNIC), 28029 Madrid, Spain; 6 CIBERSAM, ISCIII, 28029 Madrid, Spain; 7 Instituto de Investigación Sanitaria de Santiago de Compostela (IDIS), 15706 Santiago de Compostela, Spain; angel.garcia@usc.es (Á.G.); Susana.Belen.Bravo.Lopez@sergas.es (S.B.B.); 8 Center for Research in Molecular Medicine and Chronic Diseases (CIMUS), Universidad de Santiago de Compostela, 15706 Santiago de Compostela, Spain; 9 Faculty of Medicine, Universidad Complutense de Madrid, 28040 Madrid, Spain; 10 Institute of Polymer Science and Technology, CSIC, Juan de la Cierva 3, 28006 Madrid, Spain; celvira@ictp.csic.es; 11 HepatoGastro Lab., Hospital General Universitario Gregorio Marañón, 28009 Madrid, Spain; 12 CIBEREHD: ISCIII, 28029 Madrid, Spain

**Keywords:** extracellular vesicles, hydrogel, extracellular matrix, drug delivery, polyethylene glycol, cardiac regenerative therapy

## Abstract

Stem-cell-derived extracellular vesicles (EVs) have demonstrated multiple beneficial effects in preclinical models of cardiac diseases. However, poor retention at the target site may limit their therapeutic efficacy. Cardiac extracellular matrix hydrogels (cECMH) seem promising as drug-delivery materials and could improve the retention of EVs, but may be limited by their long gelation time and soft mechanical properties. Our objective was to develop and characterize an optimized product combining cECMH, polyethylene glycol (PEG), and EVs (EVs–PEG–cECMH) in an attempt to overcome their individual limitations: long gelation time of the cECMH and poor retention of the EVs. The new combined product presented improved physicochemical properties (60% reduction in half gelation time, *p* < 0.001, and threefold increase in storage modulus, *p* < 0.01, vs. cECMH alone), while preserving injectability and biodegradability. It also maintained in vitro bioactivity of its individual components (55% reduction in cellular senescence vs. serum-free medium, *p* < 0.001, similar to EVs and cECMH alone) and increased on-site retention in vivo (fourfold increase vs. EVs alone, *p* < 0.05). In conclusion, the combination of EVs–PEG–cECMH is a potential multipronged product with improved gelation time and mechanical properties, increased on-site retention, and maintained bioactivity that, all together, may translate into boosted therapeutic efficacy.

## 1. Introduction

Regenerative and reparative therapies, although they seem promising in different cardiovascular diseases, present demanding requirements that are difficult to achieve and that limit their translation into the clinical scenario [1]. Regenerative products must be biocompatible and bioactive, preferably injectable in a unique dose, and adequately retained and degraded at the site of interest. 

The main problems of cell products derive from their large variability and their lack of stability and standardization [2]. Some of the challenges seemed to be solved once it was clarified that most of their beneficial effects are caused by paracrine mediators, such as extracellular vesicles (EVs), which possess meaningful advantages as therapeutics vs. their parenteral cells [3,4]. In particular, EVs derived from cardiosphere-derived cells (CDCs) have shown potential to improve cardiac function in infarcted [5] and aged hearts [6], as well as antiarrhythmic effects [7]. However, EV retention at the target site remains as one of their main challenges [8].

The use of injectable biomaterials for cardiac regeneration, such as hydrogels, has also been explored. Those derived from cardiac extracellular matrix (cECMH) mimic the biophysical and topographical properties of the ECM and gel at physiological temperature. In addition, they improve cardiac function in preclinical models [9] and have shown safety in a phase I clinical trial [10]. However, slow gelation time, rapid degradation, and poor mechanical properties [11] are some of their physicochemical limitations. The combination of naturally derived hydrogels with synthetic materials, such as polyethylene glycol (PEG), seems promising [11] to make cECMH more suitable for therapeutic use.

Apart from providing structural support and favorable bioactivity on surrounding tissue, biomaterials can also be used for the delivery of small particles and/or cells to improve their retention at the injection site [12]. The combination of hydrogels with other bioactive products may solve some of the current limitations they possess individually, and enhance the therapeutic response with a synergistic effect [13]. However, specific designs for specific purposes will probably be needed.

The aim of this work was to develop, characterize, and evaluate in vitro and in vivo the suitability of a new product composed by EVs derived from CDCs embedded in a PEG–cECM hydrogel (EVs–PEG–cECMH) for its use as a regenerative product. Firstly, we explored the different physicochemical properties of the combined product (EVs–PEG–cECMH) vs. the cECMH alone, and how PEG, which can also be used as a purification method of EVs from conditioned medium, influenced the gelation time and the mechanical properties. Secondly, we confirmed that product combination into the EVs–PEG–cECMH did not negatively affect the bioactivity of the individual components and served to improve EV retention at the injection site compared to EV administration in the standard delivery vehicle.

## 2. Results

### 2.1. Lyophylized cECM Retained Native ECM Proteins and sGAGs

**Figure 1:**
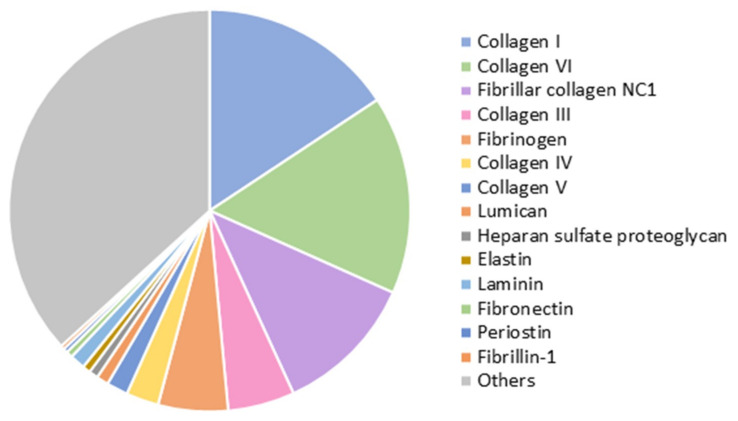
Proteins identified by liquid chromatography–tandem mass spectrometry (LC–MS/MS) in the lyophilized cardiac extracellular matrix (cECM) (*n* = 3).

Decellularization of the myocardial matrix was confirmed by the absence of visible nuclei after DAPI staining. Blyscan assay on the lyophilized samples revealed a retained sulfated glycosaminoglycan (sGAG) content of 6.1 ± 0.4 µg/mg of lyophilized cECM, of which 1.7 ± 0.6 µg/mg was O-sulfated. Liquid chromatography–tandem mass spectrometry (LC–MS/MS) confirmed the presence of around 140 different proteins related to ECM and cardiac tissue. Figure 1 shows some of the most abundant proteins identified. Collagens of different types (I, VI, III, IV, and V) compose most of the cECM, but other relevant proteins involved in providing structure and promoting cell attachment and migration, such as fibrinogen, lumican, elastin, laminin, and fibronectin, are also present. 

### 2.2. cECM Hydrogels Incorporating EVs and PEG Have Shorter Gelation Times, Larger Fiber Diameter, and Improved Mechanical Properties While Remaining Injectable and Biodegradable

#### 2.2.1. Gelation Kinetics

**Figure 2:**
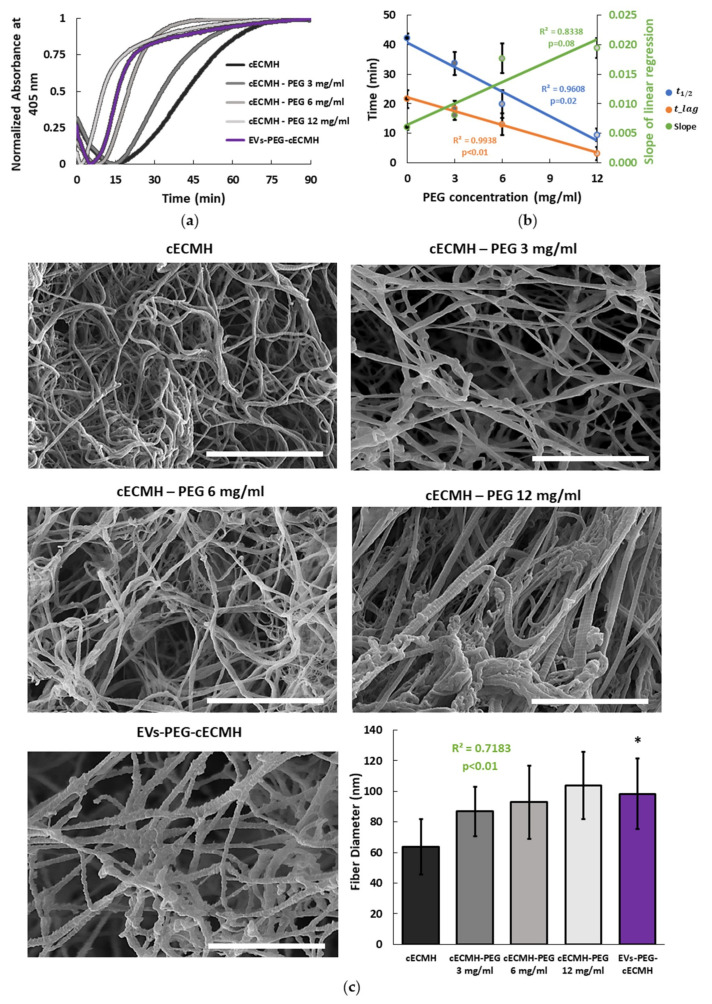
Gelation kinetics and fiber diameter. (**a**) Turbidimetric average results over time, with optical density normalized at 405 nm for the different formulations (*n* = 3, each in triplicate, for each condition). (**b**) Correlation between the PEG concentration and the half gelation time ($t_{1/2})$, the lag time ($t_{lag}$), and the slope of the linear regression (speed of gelation) obtained from turbidimetry (*n* = 3, each in triplicate, for each condition). (**c**) Scanning electron microscopy (SEM) images of the different hydrogel formulations and their average fiber diameter (*n* = 160 fibers for each condition). Scale bars correspond to 2 µm. Squared Pearson’s correlation coefficient for PEG concentration vs. fiber diameter and its significance (*p* < 0.01). * *p* < 0.001 vs. cECMH. cECMH, cardiac extracellular matrix hydrogel; PEG, polyethylene glycol; EVs, extracellular vesicles.

Turbidimetry measurements at 37 °C showed an increase in optical density (OD) as the solutions gelled. After 90 min, OD had reached a plateau in all the samples. The incorporation of PEG to the cECM solution significantly reduced the amount of time needed to reach the plateau, and, therefore, the gelation time (Figure 2a). While $t_{1/2}$ was 42 ± 2 min for the control condition, when PEG was incorporated at concentrations of 3, 6, and 12 mg/mL, the $t_{1/2}$ was reduced to 34 ± 4, 20 ± 5, and 9 ± 2 min, respectively (R^2^ = 0.96, *p* = 0.02). The reduction in $t_{1/2}$ with the incorporation of PEG was achieved due to a faster initiation of polymerization (shorter lag phase, $t_{lag}$, R^2^ = 0.99, *p* < 0.01) and to an increased speed of gelation (slope of the OD change, R^2^ = 0.83, *p* = 0.08). The relationship of $t_{1/2}$, $t_{lag}$, and the slope of gelation with the PEG concentration is summarized in Figure 2b. As PEG concentration was increased, the turbidity of the gels (final OD value without normalization) also increased (data not shown). For PEG concentrations higher than shown (16 mg/mL), no significant change in OD was observed during the 90 min, and the solution did not polymerize, even after 24 h of incubation at 37 °C. This suggested that higher PEG concentrations impede adequate gelation. 

For the combined product, EVs isolated from conditioned medium [6,7] were incorporated into the hydrogels (EVs–PEG–cECMH), observing similar gelation kinetics to hydrogels with PEG (Figure 2a). They also presented reduced lag and gelation time ($t_{1/2}$ = 13 ± 3 min) and a higher speed of gelation (slope = 0.022 ± 0.009). The estimated PEG concentration in the combined EVs–PEG–cECMH product was 8 ± 2 mg of PEG per 1 mL of cECM solution.

#### 2.2.2. Fiber Diameter

Hydrogel formulations incorporating PEG and EVs formed the characteristic nanofibrous structure with an increase in average fiber diameter, as shown and quantified in scanning electron microscopy (SEM) images (Figure 2c). Fiber density was not homogeneous across the gels, and the fibers tended to be randomly oriented in all the samples. However, fiber diameter was strongly correlated to the amount of PEG in the hydrogel (R^2^ = 0.72, *p* < 0.01). EVs–PEG–cECMH also presented an increased fiber diameter with respect to standard hydrogels (100 ± 20 nm vs. 60 ± 20 nm, *p* < 0.001).

#### 2.2.3. Injectability, Viscosity, Storage, and Loss Modulus

**Figure 3:**
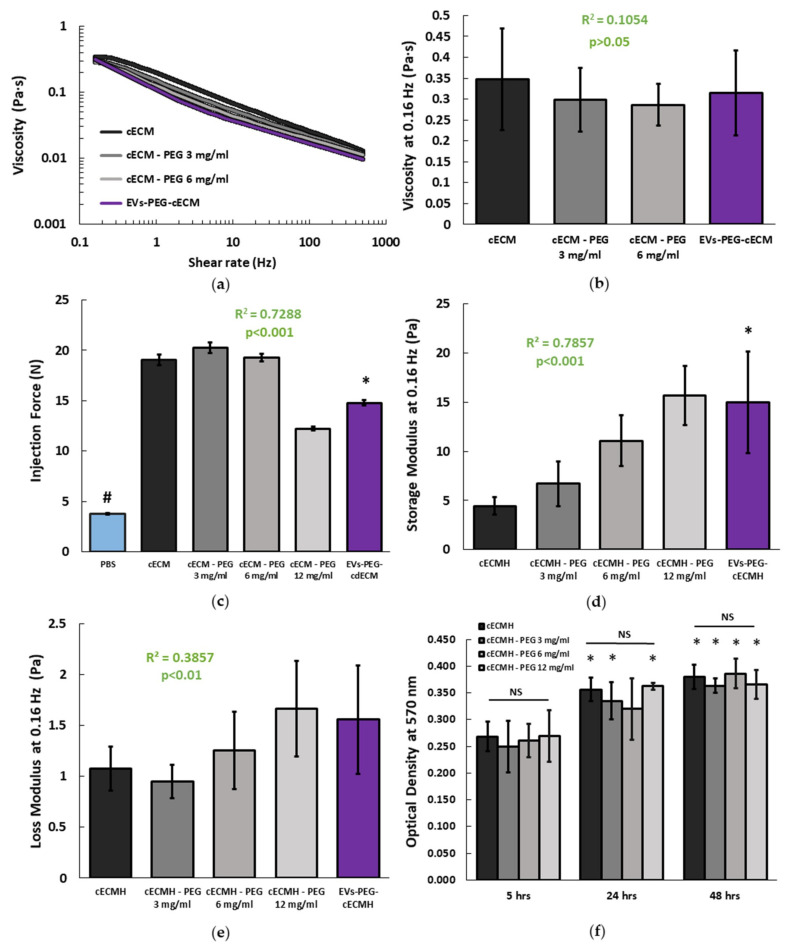
Mechanical properties and degradation of the cECMH alone, with PEG, and with PEG-isolated EVs. (**a**) Average viscosity of the different hydrogel solution formulations (*n* = 3 for each condition, each in triplicate). (**b**) Viscosity at 0.16 Hz (*n* = 3). Squared Pearson’s correlation coefficient for PEG concentration in the cECMH vs. viscosity at 0.16 Hz and its significance (*p* > 0.05). (**c**) Force required for injection (*n* = 3). Squared Pearson’s correlation coefficient for PEG concentration in the cECMH vs. injection force and its significance (*p* < 0.001). * *p* < 0.001 vs. cECMH. # *p* < 0.001 vs. all other groups. (**d**) Storage modulus at 0.16 Hz (*n* = 4). Squared Pearson’s correlation coefficient for PEG concentration in the cECMH vs. storage modulus and its significance (*p* < 0.001). * *p* < 0.01 vs. cECMH. (**e**) Loss modulus at 0.16 Hz (*n* = 4). Squared Pearson’s correlation coefficient for PEG concentration in the cECMH vs. loss modulus and its significance (*p* < 0.01) (**f**) Enzymatic degradation (soluble amines, *n* = 6, each in triplicate). * *p* < 0.05 with respect to their same group at 5 h. cECMH, cardiac extracellular matrix hydrogel; PEG, polyethylene glycol; EVs, extracellular vesicles.

All the formulations prepared remained injectable and with a viscosity suitable for their application through a Myostar injection catheter. In all the samples, the viscosity decreased as shear rate increased, as is characteristic of shear-thinning behavior (Figure 3a). This property is important for injectability, as well as for improved retention [14]. The formulation with the highest PEG concentration (12 mg/mL) presented inconsistent viscosity values (data not shown). These results seemed not to be reliable, since the very short gelation time of the samples with this concentration of PEG did not allow it to remain in soluble form during the viscosity measurement. For the remaining PEG concentrations (3 and 6 mg/mL), the amount of PEG present did not relate with a change in the viscosity of the solutions at 0.16 Hz (R^2^ = 0.1, *p* > 0.05), and the EVs-PEG–cECM did not present significant viscosity differences with respect to cECM alone (Figure 3b). Injection force measurements revealed that no additional force is required for the injection of cECM solutions incorporating PEG or the EVs–PEG–cECM compared to the injection of cECM solution alone (Figure 3c). However, the force required to inject any of the cECM solutions was significantly higher than the one required to inject the standard vehicle (*p* < 0.001, phosphate-buffered saline, PBS).

The mechanical properties of the different hydrogel formulations are summarized in Figure 3d,e. The amount of incorporated PEG was directly related to an increase in the storage modulus of the derived hydrogels (R^2^ = 0.79, *p* < 0.001). While higher PEG concentrations also lead to a significantly higher loss modulus, the correlation was weaker in comparison to the storage modulus (R^2^ = 0.39, *p* < 0.01). EVs–PEG–cECMH also presented a higher storage modulus than cECMH (15 ± 5 vs. 4.5 ± 0.9 Pa, *p* < 0.01), and a slightly higher loss modulus (1.5 ± 0.5 vs. 1.1 ± 0.2, *p* > 0.05).

#### 2.2.4. Degradation

The ninhydrin assay (indicative of the amount of soluble amines) showed that PEG incorporation into the hydrogels did not affect their degradation rate, as there were no significant differences in OD at 570 nm between any of the formulations at the same time point (*p* > 0.05, Figure 3f). All the samples presented significantly higher degradation after being incubated for 48 h with collagenase compared to 5 h of incubation (*p* < 0.05, paired Student’s *t*-tests). The combined product (EVs–PEG–cECMH) presented the highest value of OD (0.454 ± 0.08 after 5 h, 0.654 ± 0.16 after 24 h, and 0.673 ± 0.17 after 48 h) in the ninhydrin test. However, we did not consider this sample for comparison with the others because of the interference of the residual amines of the conditioned medium of the EVs in the ninhydrin test.

### 2.3. The Combined Product of EVs–PEG–cECMH Maintains the Bioactivity of the Individual Components

**Figure 4:**
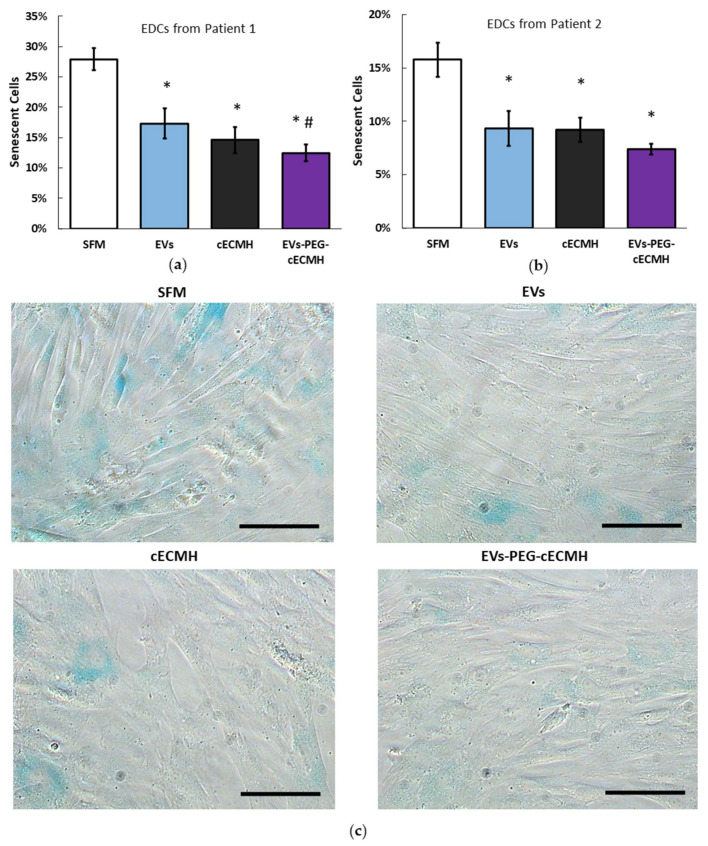
Bioactivity of EVs alone, cECMH, and EVs–PEG–cECMH, measured as anti-senescent effect. (**a**) Percentage of senescent EDCs from patient 1 under basal conditions and under exposure to EVs alone, cECMH alone, or EVs–PEG–cECMH (*n* = 3). (**b**) Percentage of senescent explant-derived cells (EDCs) from patient 2 under the different conditions (*n* = 3). (**c**) Optical microscopy images from EDCs (senescent cells in blue) from patient 1 under the different conditions. * *p* < 0.01 with respect to serum-free media (SFM). # *p* < 0.05 with respect to the EVs group. Scale bars correspond to 100 µm. cECMH, cardiac extracellular matrix hydrogel; PEG, polyethylene glycol; EVs, extracellular vesicles.

The bioactivity and potential beneficial effect of the PEG–cECMH with the embedded EVs was confirmed by evaluating the reduction in cardiac stromal cell (explant-derived cells, EDCs) senescence from two different donors in culture (this property of EVs derived from CDCs was proved in other studies [6]). As control groups for EVs–PEG–cECMH, we used EDCs incubated with serum-free media (SFM), EVs alone, or cECMH alone (Figure 4a,b). EVs and cECMH significantly reduced basal senescence (by around 40%, *p* < 0.01) in the EDCs derived from both patients. No significant differences were detected between the EVs and the cECMH used alone. When using the combined product (EVs–PEG–cECMH), basal senescence was further reduced (by ~55%, *p* < 0.001). This difference was also significant with respect to using EVs alone in one of the patients (*p* < 0.05). Images taken with optical microscopy in EDCs from patient 1 show a higher proportion of senescent EDCs (in blue) under basal conditions (SFM) than when exposed to EVs alone or cECMH alone, and a lower proportion of senescent cells when exposed to EVs–PEG–cECMH (Figure 4c).

### 2.4. The Combined Product of EVs–PEG–cECMH Shows a Higher Local EV Retention In Vivo

#### 2.4.1. EV Release

**Figure 5:**
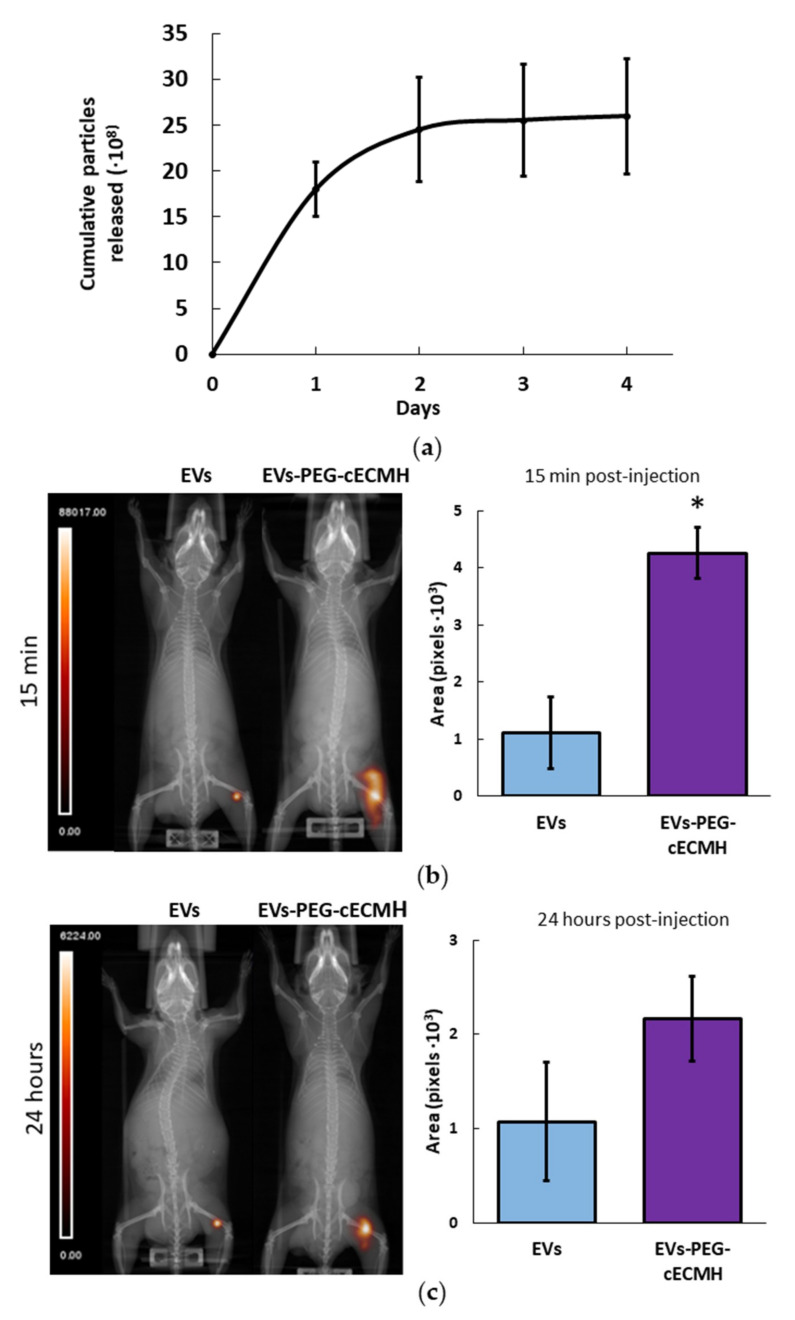
EV release from the EVs–PEG–cECMH and EV in vivo retention. (**a**) Number of cumulative EVs released into the medium from EVs–PEG–cECMH for 4 days (*n* = 3, each run in triplicate), evaluated using nanoparticle tracking analysis (NTA). The error bars were obtained by the standard deviation of the three different samples. After 48 h there were no significant differences (*p* > 0.05) with the number of particles detected in control samples. (**b**) Representative single-photon emission computed tomography–computed tomography (SPECT–CT) images and area of radioactively labeled EVs (*n* = 3) after in vivo injection of EVs resuspended in phosphate buffered saline (PBS) or in PEG–cECM (EVs–PEG–cECM) 15 min and (**c**) 24 h post-injection. * *p* < 0.05. cECMH, cardiac extracellular matrix hydrogel; PEG, polyethylene glycol; EVs, extracellular vesicles.

Most EVs incorporated into the PEG–cECMH were cumulatively released during the first 48 h (Figure 5a). After this time, there were no significant differences (*p* > 0.05) between the particles detected in control samples (EVs resuspended in SFM) and the cumulative number of particles released by the EVs–PEG–cECMH. During days 3 and 4, some particles continued to be released, but in considerably lower amounts. The number of particles detected in SFM alone (without EVs) or in medium exposed to cECMH or PEG–cECMH alone (without EVs embedded) was insignificant, confirming that particles detected in control samples and in samples from EVs–PEG–cECMH did correspond to the added EVs.

#### 2.4.2. In Vivo Retention

Single-photon emission computed tomography–computed tomography (SPECT–CT) images of mice revealed a larger area of ^99m^Tc radioactively labeled EVs at the subcutaneous injection site when administered embedded in the PEG–cECMH compared to their administration resuspended in PBS (Figure 5). The area of EVs was four times larger (*p* < 0.05) after 15 min post-injection when using the PEG–cECMH to deliver them. After 24 h, the average area of concentrated EVs was twice as large when applied with the PEG–cECMH, but the difference was not significant (*p* = 0.1).

## 3. Discussion

According to our results, the combination of EVs–PEG–cECMH optimizes the features of its individual bioactive components and makes it more suitable in different therapeutical applications. EVs–PEG–cECMH maintained or significantly improved the physicochemical properties (particularly the gelation time), while not hindering injectability and degradation vs. cECMH alone. PEG at low concentrations, which, in fact, can be used to isolate EVs from conditioned medium, was responsible for these differences. In addition, the EVs are progressively released from the EVs–PEG–cECMH and are better retained at the injection site in vivo when administered in the EVs–PEG–cECMH compared to EVs administered in the standard vehicle (PBS). The combination of the products reduces cellular senescence, maintaining the bioactive properties of cECMH and EVs alone.

Although the incorporation of low-molecular-weight PEG up to a certain concentration (12 mg/mL) proportionally improved the gelation properties of the cECMH, higher PEG concentrations impeded gelation. cECMH have collagen as their main component, so they share some similarities in the gelation kinetics with collagen hydrogels [18]. During the lag phase, collagen nucleation occurs by forming triple helices. Later, during the gelation phase, these assemble into ordered structures to form fibrils [19]. The addition of PEG seems to speed up the nucleation phase and favor collagen fibril formation during gelation. This more rapid formation could be responsible for the larger fiber diameter [20] and the higher turbidity of the gels [19]. Larger fiber diameter could also result from PEG entangled in collagen fibers, similar to hyaluronic acid chains incorporated into a collagen network without crosslinking [21]. Other studies have incorporated PEG into cECM or collagen hydrogels to tailor their material properties [20,22]. However, these studies incorporate multi-armed PEG with functionalized groups that crosslink with the collagen network or modified PEGs that require external triggers for polymerization, such as UV light. The addition of functionalized multi-armed PEGs at high concentrations (12–24 mg/mL) still allows the formation of gels at 37 °C without external triggers and the tuning of mechanical properties and degradation, but they do not improve the gelation time [20]. Slow gelation times, which can increase tissue necrosis, have been highlighted as a main drawback of ECM hydrogels [11]. 

Mechanical properties of the ECM hydrogels are also improved with the incorporation of PEG, while injectability and biodegradation are maintained. The mechanical properties of cECMH alone are considered insufficiently robust for providing prolonged mechanical support in the injured heart, where they are subjected to significant strain and contraction [11]. In addition, these properties influence cell fate [23] and migration [24]. PEG–cECMH or EVs–PEG–cECMH present a significantly higher storage modulus, making them more suitable for cardiac applications than cECMH alone. The reduction in the gelation time and the increase in the elasticity of the cECMH after gelation did not negatively affect the viscosity of the liquid form and their injectability through the Myostar catheter. Even the solutions with reduced gelation time could be uniformly injected and did not clog the catheter, an essential property for cardiac applications [25]. In fact, when adding the EVs, the force required for injection was considerably lower. Moreover, the incorporation of PEG with the method presented here (linear PEG without functionalized groups that react with amines in collagen) does not influence the cECMH biodegradation rate, which in vivo studies have shown to completely degrade within 14–28 days post-injection [9].

Hydrogels as vehicles to deliver EVs have already shown potential for improved in vivo retention and boosted cardiac function [15,16,17]. Applications where fast gelation time becomes especially important (e.g., to retain bioactive molecules at the site of injection [11], embed cells, or create 3D tissue-like structures [26]) while maintaining injectability and natural tissue environment may potentially benefit from the improved physicochemical properties of PEG incorporation into ECM hydrogels. The combined product presented here (EVs–PEG–cECMH), with an estimated PEG concentration of 8 ± 2 mg, (matching the results obtained in the physicochemical analysis, in which the combined product presented average values between the cECMH with PEG at 6 and 12 mg/mL), benefits from these improved physicochemical properties. 

EVs administered in vivo in the PEG–cECM solution also achieved the goal of improved retention at the target site when compared to EVs administered with the standard vehicle (PBS). This is probably because the fast gelation time of the combined product prevents the rapid absorption of the subcutaneously injected EVs into the bloodstream right after the injection. According to nanoparticle tracking analysis (NTA) measurements, EVs encapsulated in the PEG–cECMH are mostly released during the first 48 h, which is in line with other studies [27]. In addition, SEM images of the EVs–PEG–cECMH show rougher collagen fibers with respect to cECMH or PEG–cECMH alone. This phenomenon has also been observed in other studies, where this roughness has been related to embedded proteins and vesicles [28]. After several days of soaking the hydrogels, as the particles were released to the medium, the fibers started to look smoother. Accordingly, our in vivo experiment showed that, although a high proportion of the EVs are probably released from the PEG–cECMH during the first day, a large amount of EVs still remain encapsulated at the injection site after 24-hours. 

Both the cECMH [9] and the EVs [5] individually have shown favorable effects for the treatment of cardiac pathologies in preclinical models. While EVs have been shown to halt proinflammatory and profibrotic pathways and induce angiogenesis [5], the ECM hydrogels provide mechanical support and an environment with a structure and protein composition close to the native cardiac tissue [9]. Moreover, ECM hydrogels promote cellular influx and their degradation products favor cellular migration, proliferation [29], and angiogenesis [30]. In this study, despite EVs and the cECMH individually showing significant (and similar) anti-senescent properties in cardiac stromal cells from different human donors, this effect tended to be enhanced when both products were used together, indicating a possible synergistic effect of both products. 

Despite the EVs embedded in PEG–cECMH showing promising characteristics for its use in cardiac regenerative applications, future studies should confirm if the improved physicochemical properties of the combined product (provided by the PEG), the bioactivity, and the enhanced in vivo EV retention vs. the use of the individual components alone translate into improved functionality in an animal model. Furthermore, mechanical properties of the PEG–cECMH, although improved with the addition of PEG, are still far from mimicking that of the native ECM. In this study, EVs were tracked in vivo only for 24 h, and our in vitro tests demonstrated that most particles are released during the first 48 h. Presumably, a longer liberation time of the EVs from the PEG–cECMH could increase the efficacy of the product, mimicking repeated dosing. Labeling the EVs with other molecular methods (such as with optical probes), would allow in vivo tracking of the EVs for longer. This could help determine for how long the EVs can remain at the target site after being injected in the PEG–cECMH. To further improve and optimize these properties, it becomes necessary to explore modifications in the hydrogel composition. For example, modifying the ECM concentration and incorporating additional synthetic materials could contribute to the tailoring of mechanical and degradation properties [18,20], as well as pore size [31] and EV release rate.

Regardless of the limitations cited above, here we show that the delivery of EVs isolated with PEG and embedded in cECMH offer a minimally invasive, injectable therapeutical product with a fast thermosensitive response at physiological temperature. The product can provide an environment with proteins naturally present in the myocardium and with mechanical and structural support, while it enables the retention and release of the EVs at the target site. The combined product (EVs–PEG–cECMH) presents bioactivity and is biodegradable. As EVs–PEG–cECMH solves some of the current limitations of this type of biological therapy, it can potentially be used in different regenerative medicine applications. 

## 4. Materials and Methods

### 4.1. EDCs, CDCs, and Derived Extracellular Vesicle Isolation

EVs used as therapeutic product were isolated from human CDCs, as previously described [6,7]. Briefly, cardiac biopsies from patients undergoing cardiac surgery were processed to obtain 1–2 mm explants. EDCs colonized the plate after approximately 16 days. EDCs could be further cultured on ultra-low-attachment plates for 72 h to form cardiospheres. After three further passages in standard plates, CDCs were left in FBS-free IMDM (SFM) for 15 days to secrete EVs into the medium. The conditioned medium was filtrated using a 0.45 µm filter and ultraconcentrated with Centricon-Plus 70 Centrifugal Filter with 3-kDa cut-off frequency. EVs were quantified by NTA ( Nanosight NS300, Malvern Paranalytical, Malvern, UK) and with Bradford assay after lysis and protease inactivation. The mean EV size obtained by the NTA software was 170 nm ± 20 nm, with a maximum particle size of 450 nm. For experiments, EVs were incubated overnight at 4 °C in 4% *w/v* polyethylene glycol (PEG), precipitated by centrifugation at 1500× *g* for 30 min, and resuspended in the corresponding medium (SFM or cECMH) at a concentration of 5 mg/mL, unless otherwise stated. Considering the 4% *w/v* PEG concentration in the conditioned medium, the size of the pellet containing the EVs (150–250 µL) and the volume of cECM in which the pellet was resuspended (1 mL), the estimated PEG concentration in the combined product of EVs–PEG–cECM solution was 8 ± 2 mg/mL. 

### 4.2. Porcine Myocardial Matrix Decellularization, Lyophilization, and Characterization

Porcine myocardial matrix was decellularized with slight modifications from a previous protocol from our group [32]. The heart from six euthanized minipigs (6.5 months old on average, 3 males) were immediately extracted, cleaned from fat, valves, and fibrotic regions, and the myocardium was cut into thin slices (1 mm thick). The slices were individually decellularized in a distilled water solution at 1% *w/v* of sodium dodecyl sulfate (SDS) under constant shaking for 48–72 h, and then washed in distilled water for another 72 h. All the solutions were changed daily. The slices were then disinfected for 30 min in peracetic acid at 1% and kept in phosphate-buffered saline (PBS) with 1% penicillin/streptomycin (Gibco) at 4 °C until lyophilization. Three slices from different animals (decellularized and non-decellularized) were frozen in Tissue O.C.T., cut into 4 µm slices, and stained with DAPI to confirm decellularization. This decellularization protocol has been previously shown in our group to yield less than 50 ng of DNA content per mg of tissue [32]. The lyophilized samples (in a Telstar, Lioalfa-6 lyophilizer, Munich, Germany) were later cut into small pieces (around 1 mm size), mixed, and stored at −20 °C until used for hydrogel preparation. sGAG content (total and O-sulfated) and proteins present in the lyophilized matrix were confirmed in 3 samples by using the Blyscan assay (Biocolor Ltd., Carrickfergus, UK) and by LC–MS/MS (in a Triple TOF 6600, Sciex, Old Connecticut Path Framingham, MA, USA), respectively. 

The LC–MS/MS analysis was made using a previously standardized method by our group [33,34]. An equal amount of protein from the samples was loaded on a 10% SDS-PAGE gel. The run was stopped as soon as the front had penetrated 3 mm into the resolving gel [35,36]. The protein band was visualized by Sypro Ruby fluorescent staining (Lonza, Basel, Switzerland), excised, and subjected to in-gel, manual tryptic digestion following the protocol described previously by our group [33,34]. Peptides were extracted by performing three 20-min incubations in 40 μL of 60% acetonitrile dissolved in 0.5% HCOOH. The resulting peptide extracts were pooled, concentrated in a SpeedVac, and stored at −20 °C.

Digested peptides were separated using reverse phase chromatography. Gradient was developed using a micro liquid chromatography system (Eksigent Technologies nanoLC 400, Sciex, Framingham, MA, USA) coupled to a high-speed Triple TOF 6600 mass spectrometer (Sciex, Framingham, MA, USA) with a micro flow source. The analytical column used was a silica-based reversed phase column YMC-TRIART C18 150 × 0.30 mm, with a 3 mm particle size, and 120 Å pore size (YMC Technologies, Teknokroma, Sciex, Framingham, MA, USA). The trap column was a YMC-TRIART C18 (YMC Technologies, Teknokroma, Sciex, Framingham, MA, USA) with a 3 mm particle size and 120 Å pore size, switched on-line with the analytical column. The loading pump delivered a solution of 0.1% formic acid in water at 10 µL/min. The micro-pump provided a flow rate of 5 µL/min and was operated under gradient elution conditions, using 0.1% formic acid in water as mobile phase A, and 0.1% formic acid in acetonitrile as mobile phase B. Peptides were separated using a 90-minute gradient ranging from 2% to 90% mobile phase B (mobile phase A: 2% acetonitrile, 0.1% formic acid; mobile phase B: 100% acetonitrile, 0.1% formic acid). Injection volume was 4 µL. 

Data acquisition was carried out in a TripleTOF 6600 System (Sciex, Framingham, MA, USA) using a data-dependent workflow. Source and interface conditions were as follows: ion spray voltage floating (ISVF) 5500 V, curtain gas (CUR) 25, collision energy (CE) 10, and ion source gas 1 (GS1) 25. The instrument was operated with Analyst TF 1.7.1 software (Sciex, Framingham, MA, USA). Switching criteria were set to ions greater than the mass to charge ratio (*m/z*) 350 and smaller than *m/z* 1400, with charge state of 2–5, mass tolerance 250 ppm, and an abundance threshold of more than 200 counts (cps). Former target ions were excluded for 15 s. The instrument was automatically calibrated every 4 h using as external calibrant tryptic peptides from pep Cal Mix (Sciex, Framingham, MA, USA).

After MS/MS analysis, data files were processed using ProteinPilotTM 5.0.1 software from Sciex, which uses the algorithm ParagonTM for database search and ProgroupTM for data grouping. Data were searched using a Sus Scrofa specific Uniprot database. False discovery rate was performed using a non-lineal fitting method, displaying only those results that reported a 1% global false discovery rate or better [37,38]. For the plot in Figure 1, relative abundance of each protein was determined from the number of peptides identified, corrected by the size of the protein (in KDa, from Uniprot).

### 4.3. Cardiac Extracellular Matrix Hydrogel (cECMH) Synthesis 

Hydrogels from the lyophilized decellularized cECM were prepared fresh for each experiment, with slight modifications from previous protocols [39]. The lyophilized myocardial matrix was solubilized with pepsin (P6887, Sigma-Aldrich, at 1 mg/10 mg of lyophilized matrix) in 0.01 M HCl (1 mL/10 mg of matrix) under constant stirring for 72 h, at which point no solid particles were visible. The solubilized cardiac matrix solution was placed on ice and adjusted to pH 7.4 by progressive addition of 0.1 M NaOH, and to physiological salt concentration (PBS 1x) by addition of 1/9 of the volume of PBS 10x. For hydrogel formulations incorporating PEG, (molecular weight = 8000, P5414, Sigma-Aldrich, at 3, 6, 12, or 16 mg/mL in the final solution), the corresponding amount of polymer was dissolved in the PBS 10x, sterile filtered, and added to the solution. All formulations were brought to a final concentration of 8 mg of solubilized cardiac matrix per mL of solution with the addition of PBS 1x. The solution was centrifuged at 4000 rpm and 4 °C for 5 min to remove any remaining insoluble particles (if any) and kept at 4 °C until used (maximum 24 h).

### 4.4. Gelation Kinetics

Gelation kinetics from three different batches of the control hydrogels, with PEG at 3, 6, 12, and 16 mg/mL, and with EVs prepared as described above, were studied by turbidimetry as previously published [18,28]. A total of 100 µL of each solution in triplicate were placed in a 96-well plate, and the OD at 405 nm and 37 °C was measured every 15 s with a SynergyTM HTX Multi-Mode Microplate Reader (BioteK, Winooski, VT, USA) during 90 min (when a plateau in all samples had been reached). OD values were averaged and normalized for each group. From the normalized plot, the half gelation time ($t_{1/2}$, at which normalized OD was 0.5), the lag phase ($t_{\mathrm{lag}}$, at which the linear fit in the linear region of the plot was zero), and the speed of gelation (S, slope of the linear fit) were compared among the different groups.

### 4.5. Rheometry and Injectability

Viscosity and rheology measurements were performed in a TA Instruments AR-G2 rheometer (New Castle, DE, USA) on samples with the different conditions (cECMH, and cECMH with PEG at 3, 6, and 12 mg/mL, and with EVs). Viscosity was measured with a 40 mm diameter aluminum Peltier plate at the different conditions (from three different batches, each sample in triplicate). The rheometer was maintained at 25 °C and viscosity was measured between 0.1 and 500 Hz. For storage (G’) and loss modulus (G’’) measurements, 1500 µL of hydrogel solution was gelled for 24 h at 37 °C on 25 mm diameter disc-shaped molds. The linear viscoelastic region was determined in 3 samples, applying an oscillatory strain sweep with amplitudes from 0.01 % to 200% at a frequency of 1 Hz using a 25 mm diameter sand-blasted Peltier plate. In 4 samples of each condition (from two different batches), the dynamic frequency sweep at a fixed strain within the viscoelastic region (0.15%) was performed from 0.01 to 2 Hz at 37 °C. G’ and G’’ at 1 rad/s (0.16 Hz) were plotted. 

Injection forces are also important parameters for shear-thinning hydrogel characterization [14]. Injectability of the different preparations was confirmed through a commercially available Myostar injection catheter equipped with a 27 G needle at the tip (Biosense Webster, Irvine, CA, USA). The force required for injecting the different solutions with a 1 mL Luer-lock syringe connected to the Myostar catheter was measured at an injection speed of 500 µL/min using a QTest 1 L Elite System (MTS, Artisan Technology Group, Champaign, IL, USA) and recorded using TestWorks Software. Measurements were performed at room temperature in 3 samples for each group (PBS, cECMH, cECMH with PEG at the different concentrations, and EVs–PEG–cECMH).

### 4.6. Scanning Electron Microscopy

To study the effect of PEG and EVs on the hydrogel structure and fiber diameter, SEM images from hydrogels of two different batches at the different conditions (cECMH, cECMH with PEG at 3, 6, and 12 mg/mL, and EVs–PEG–cECMH) were taken. The solubilized matrix (800 µL) was gelled at 37 °C for 24 h, and then dehydrated with a series of ethanol washes (2 h at 20%, 2 h at 40%, overnight at 60%, 2 h at 80%, 2 h at 100%, and overnight at 100%). The dehydrated samples were dried in a Thar R100W reactor by using supercritical CO_2_, as previously described [28], and then were immersed in liquid nitrogen before sectioning. Sectioned samples were mounted and sputter coated for 75 s with gold using Leica EM ACE600 (Wetzlar, Germany) to prepare them for imaging with a Philips XL30 SEM (Eindhoven, The Netherlands). Four random images at 24,000x were taken for each sample, and the widths of 20 distinguishable fibers per image (160 fibers per condition) were measured using ImageJ Software.

### 4.7. Enzymatic Degradation Assay

The degradation assay of the hydrogels from six samples (coming from three different batches) at the different conditions (cECMH, and cECMH with PEG at 3, 6, and 12 mg/mL) was performed as previously described, with slight modifications [20]. Briefly, the solubilized matrix (20 µL) was gelled at 37 °C for 24 h in a 1.5 mL Eppendorf. A total of 20 µL of collagenase type II (200 units/mL, LS004176, Worthington) dissolved in 0.1 M Trizma base buffer (T1503, Sigma-Aldrich) pH 7.4 and 0.25 M CaCl_2_ were added, and the samples were further incubated at 37 °C for 5, 24, and 48 h. A total of 20 µL of collagenase in 20 µL of PBS was used as blanks. Following incubation, the samples were centrifuged at 15000 rpm for 5 min, and 10 µL of the supernatant was mixed with an equal volume of 2% ninhydrin reagent solution (N7285, Sigma-Aldrich). The samples were boiled at 10 min in a water bath, and then 380 µL of distilled water was added. In total, 100 µL (in triplicate) was transferred into a 96-well plate and the OD at 570 nm was measured using an EMax^®^ Plus Microplate Reader (BioteK, Winooski, VT, USA). With the ninhydrin assay, a higher OD at 570 nm is indicative of more soluble amines.

### 4.8. EV Bioactivity—Antisenescent Effect

The bioactivity of the EVs, the cECMH, and the PEG–cECMH with the encapsulated EVs (EVs–PEG–cECMH) was tested by investigating their anti-senescent effect in EDCs coming from two patients different from the EVs used as treatment. In triplicate, 250 µL samples of EVs (at 0.56 mg/mL) in SFM, cECM alone, or EVs in PEG–cECM were incubated for gelation during 24 h at 37 °C in 12-well Transwells^®^ (3460, Corning^®^). EDCs from passage 3 were seeded at a concentration of 15,000 cells/cm^2^ in fibronectin precoated 12-well plates. The cells were left for 24 h to attach, and then the medium was changed to SFM (2 mL/well), and the Transwells^®^ with SFM (controls), EVs in SFM, cECMH, or EVs–PEG–cECMH were added. After 72 h, the Transwells^®^ were removed and the cells fixed and stained with the senescence-associated beta-galactosidase assay, following the manufacturer’s instructions (ab65351 Senescence Detection Kit, abcam^®^). A total of 14 images at 20x (around 650 cells) per well were taken with a Leica DMI3000B optical microscope and Leica DFC310 FX camera (Wetzlar, Germany), and analyzed using ImageJ Software. Cells were classified as senescent or non-senescent depending on whether they presented a blue color, and the percentage of senescent cells was calculated.

### 4.9. EV Release from cECMH

Three samples of 100 µL of solubilized cECM and with the EVs resuspended in PEG–cECM were incubated for gelation during 24 h at 37 °C in 24-well Transwells^®^ (3422, Corning^®^). EVs resuspended in SFM were used as controls. The samples were later placed in 24-well plates, where 600 µL of SFM was added to each well. The medium was collected daily and replaced, and the number of particles released was measured in triplicate by NTA using a Nanosight NS300 instrument and Nanosight NTA 3.4. software (Malvern Paranalytical, Malvern, UK) [40]. The samples were injected at room temperature into the sample chamber using a syringe pump, and, for each sample, three videos of 60 s were recorded with screen gain 1 and camera level at 11.

### 4.10. EV In Vivo Retention

#### 4.10.1. EV Labeling

Radioactive labeling of the isolated EVs was carried out with the SPECT radionuclide ^99m^Tc based on previous protocols of exosome radiolabeling [41]. Briefly, 9–12 mCi of commercial [^99m^Tc] NaTcO_4_ (Curium Pharma, Madrid, Spain) was reduced in the presence of 0.01M SnCl_2_ in HAc (10%). The reaction was performed for 5 min at 37 °C and 700 rpm, under N_2_ atmosphere. Then, the mixture was neutralized (pH = 7) with 2.8 N NaOH. EVs (3 mg, 400 µL) were added to the ^99m^Tc (IV) solution, and incubated and shacked for 30 min at 37 °C and 700 rpm. Radiolabeled EVs were purified by centrifuging with 10 KDa Amicon filters (Merck Life Science, Darmstadt, Germany) and resuspended in 400 µL of PBS or PEG–cECM solution. Radiochemical purity of the recovered product was established by radio-thin-layer chromatography (iTLC) using a miniGita Single system (Elisa-Raytest, Angleur, Belgium).

#### 4.10.2. EV Tracking by SPECT-CT Imaging

Multimodality imaging was performed with a small-animal SPECT scanner (µSPECT, MILabs, Houten, the Netherlands) and a preclinical CT system (Super Argus, SEDECAL, Algete, Spain). SPECT and CT images were acquired 15 min and 24 h after subcutaneous administration of an average 300 µCi dose (no significant differences between groups) of radiolabeled EVs (3 mg of protein, 400 µL of PBS or PEG–cECM solution). Six Balb/C, 15-week-old female mice of 23.3 ± 1.8 g were divided in two groups; half of them (*n* = 3) received EVs in PBS and the rest EVs in PEG–cECM. To co-register the SPECT and CT images, each animal was placed on an in-house multimodal bed surrounded by three noncoplanar capillaries filled with a mixture of ^99m^Tc and Iopamiro (Bracco Imaging S.p.A, Milan, Italy), which was visible in both modalities. The SPECT acquisition parameters were an isotropic voxel size of 0.75 mm and an acquisition time of 15 min and 1.25 h. SPECT images were reconstructed using two-dimensional ordered subset expectation maximization (OSEM-2D) with 16 subsets and 1 iteration. The CT was obtained immediately after completion of SPECT imaging. CT study was acquired using an X-ray beam current of 240 mA and a tube voltage of 40 kVp, and reconstructed using an FDK algorithm [42]. SPECT-CT images were co-registered following the method of García-Vazquez V. [43].

#### 4.10.3. Image Analysis

The area of radioactivity was segmented on the SPECT images using a threshold at 50% of the maximum using ImageJ 1.49i. The area was compared between the animals that had been injected with the radioactively labeled EVs resuspended in PBS vs. EVs resuspended in the PEG–cECM. 

### 4.11. Statistical Analysis

Results are presented as mean ± standard deviation in the text and in figures. Continuous variables were compared using Student’s *t*-tests. Pearson tests were performed to study the correlation between the different parameters and the PEG concentration in the cECMH.

## 5. Conclusions

Hydrogels derived from cardiac extracellular matrix (cECMH) incorporating polyethylene glycol (PEG) present promising characteristics for the delivery of extracellular vesicles (EVs) for different regenerative applications. Once injected into the target tissue, the product rapidly gels at physiological temperature. The incorporation of EVs with PEG to the cECMH improves the mechanical properties while maintaining the injectability and the biodegradability. In addition, the combined product showed bioactivity similar to its individual components. With the EVs–PEG–cECMH, the EVs are better retained in vivo on-site. These improved properties of the combined product solve some of the current limitations of the individual use of these regenerative components, which may be translated into an increased therapeutic efficacy.
